# Spike Glycoprotein-Mediated Entry of SARS Coronaviruses

**DOI:** 10.3390/v12111289

**Published:** 2020-11-11

**Authors:** Lin Wang, Ye Xiang

**Affiliations:** Center for Infectious Disease Research, Beijing Frontier Research Center for Biological Structure & Beijing Advanced Innovation Center for Structural Biology, Department of Basic Medical Sciences, School of Medicine, Tsinghua University, Beijing 100084, China; wanglin_peacefultree@126.com

**Keywords:** SARS coronaviruses, COVID-19, spike glycoprotein, entry, receptor binding, conformational change, membrane fusion

## Abstract

Severe acute respiratory syndrome coronavirus (SARS-CoV) and SARS-CoV-2 are enveloped, positive-sense, single-stranded RNA viruses and causes of epidemic diseases that have resulted in public health emergencies worldwide. Angiotensin-converting enzyme 2 (ACE2) is the receptor that allows the entry of these two viruses into host cells, a key step in the life cycle of the pathogens. The characterization of the interactions of ACE2 with the viral spike glycoproteins and structural studies of the ACE2-binding-induced conformational changes in the viral spike glycoproteins have furthered our understanding of the entry processes of these two viruses, and these studies provide useful information that will facilitate the development of antiviral agents and vaccines to control the diseases.

## 1. Introduction

Seventeen years after the short outbreak of the severe acute respiratory syndrome (SARS) in 2003, another coronavirus-related epidemic disease, coronavirus disease 2019 (COVID-19) has now spread globally and has affected the lives of almost everyone on this planet. SARS coronavirus (SARS-CoV) and SARS-CoV-2 are the etiological agents of SARS and COVID-19, respectively. These two enveloped, positive-sense, single-stranded RNA viruses are members of the family *Coronaviridae*, which belongs to the order *Nidovirales* [1,2,3,4]. Viruses HCoV-NL63, HCoV-229E, HCoV-OC43, and HCoV-HKU1 are members of the same family and are also human pathogens but usually cause only mild upper respiratory tract infections [5,6,7]. The *Coronaviridae* family is comprised four genera: *Alphacoronavirus*, *Betacoronavirus*, *Gammacoronavirus*, and *Deltacoronavirus*. SARS-CoV and SARS-CoV-2 belong to the *Betacoronavirus* genus [8,9,10]. The SARS-CoV and SARS-CoV-2 particles are roughly spherical with a diameter of 80–120 nm. Protruding spikes are decorated on the surface of the viral particles. The viral spike (S) glycoprotein plays an essential role in mediating the entry of the virus into host cells. In addition to the S glycoprotein, the membrane protein (M) and the small envelope protein (E) are also embedded in the virus envelope. The nucleocapsid (N) protein and the RNA genome of 26.2–31.7 thousand nucleotides form a helical ribonucleocapsid (RNP) complex that is encapsulated in the envelope [11,12].

SARS-CoV and SARS-CoV-2 are transmitted mainly via direct contact, air droplets, or aerosols [13]. Once attached to the mucosal surface, such as those of the respiratory tract or eyes, the viruses enter cells through their exclusive receptors and other required components. Specific interactions between the viral surface spike protein and the cell-surface receptor anchor the virus onto the surface of the host cell. The membrane barrier of the host cell is then overcome by the receptor-mediated fusion of viral and cell membranes. After membrane fusion, the viral genome is released into the cytoplasm, where replication is initiated to produce thousands of progeny viruses.

Angiotensin-converting enzyme 2 (ACE2) was identified as a functional receptor for SARS-CoV shortly after the outbreak of SARS in 2003 [14]. ACE2 binds the SARS S glycoprotein with high affinity [15]. Ectopic expression of human ACE2 renders cells and animals susceptible to SARS-CoV infection [14,16]. Antibodies targeting ACE2 can block SARS-CoV infection [14]. Early in 2020, this same protein was identified as the major receptor for SARS-CoV-2 [8,17]. Given the high sequence homology between the receptor binding domains of these two viruses (72.2% identity among 180 residues), the finding was not a complete surprise [17,18]. In vitro data showed a much higher binding affinity of ACE2 to the SARS-CoV-2 spike protein (Kd of ~15 nM) compared to that of SARS-CoV (Kd of ~326 nM) [19]. This high-affinity binding to ACE2 is believed to be one of the reasons for the high infection efficiency of the new virus.

Receptor-mediated entry is a key step in the life cycle of viruses and the major target of our adaptive immune systems. Understanding the entry mechanisms of SARS coronaviruses will provide useful information for the development of vaccines and effective antiviral therapies. Here, we briefly summarize results of recent studies of SARS coronaviruses and hope that this short summary of relevant studies will help in the fight against COVID-19.

## 2. The Spike Glycoprotein

**Figure 1:**
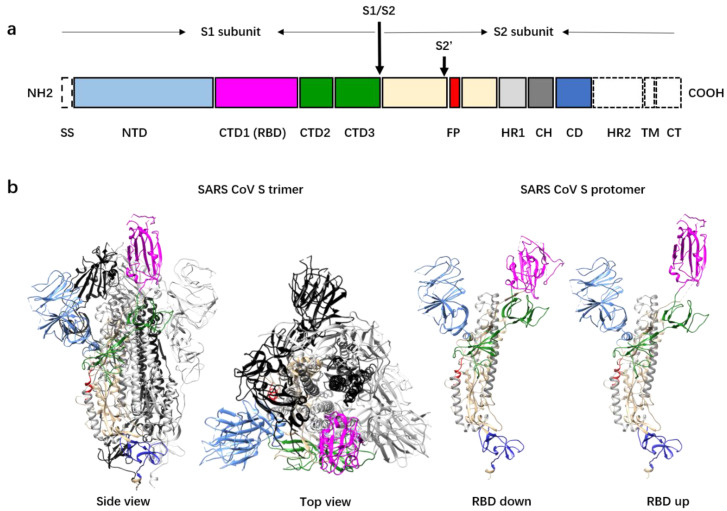
Domain organization and pre-fusion structure of the severe acute respiratory syndrome coronavirus (SARS-CoV) spike (S) protein. (**a**) A schematic diagram showing the primary sequence and domain organization of the SARS-CoV S protein. The domains and subunits of S are indicated and highlighted with different colors. Domains not shown in the 3D structure (in panel **b**) are colored white. SS, signal sequence; NTD, N-terminal domain; CTD1/2/3, C-terminal domains 1/2/3; RBD: receptor binding domain; S1/S2: S1/S2 protease cleavage site; S2′: S2′ protease cleavage site; FP: fusion peptide; HR1: heptad repeat 1; CD: connector domain; HR2: heptad repeat 2; TM: transmembrane domain; CT: cytoplasmic tail. (**b**) Left panel: Pre-fusion structure of the SARS-CoV S protein trimer with one RBD in the “up” conformation shown in side view and top view. The protomer with the “up” RBD is shown in ribbons colored as in the schematic diagram (panel **a**). The other two protomers with the “down” RBDs are colored black and gray. Right panel: Ribbon diagrams showing one RBD “down” protomer and one RBD “up” protomer.

The S protein of coronavirus is a type I viral membrane fusion protein and the key for coronavirus entry into host cells [3,20,21]. The coronavirus S protein is synthesized as a precursor protein of between 1200 and 1300 amino acids, which is cleaved by host proteases into two subunits, S1 and S2, during biogenesis or virus assembly (Figure 1a) [22,23,24,25]. The S glycoproteins assemble to form mushroom-like homotrimers on the surface of viral particles (Figure 1b). The S2 subunit structure is composed of an ecto helix core of three helixes, a transmembrane domain of a single helix, and a short intracellular domain of approximately 39 amino acids. Nine helixes from the three S2 protomers form a nine-helix bundle that constitutes the stem of the mushroom. Further cleavage of the S2 subunit at the S2′ cleavage site is essential for virus entry [26,27]. The S2′ cleavage site is approximately 130 residues downstream from the N terminus of the S2 subunit. Immediately after the S2′ cleavage site is a hydrophobic peptide fragment predicted to be the fusion peptide [28,29]. This fusion peptide, once exposed, is inserted into the host cell membrane and is crucial for virus entry. The S1 structure involves four distinct domains that wrap around the top of the helix bundle and form the head of the mushroom. The N-terminal domain (NTD) is located at the outer rim of the mushroom head, whereas the C-terminal domain 1 (CTD1) covers the distal top of the helix bundle. The CTD1s of most coronaviruses that infect humans are responsible for receptor recognition and binding [30,31]. The NTDs of S1 are believed to be involved in the attachment of the virus onto the host cell surface mediated by polysaccharides such as these with a sialic acid at the distal end [32]. The NTDs of some of the coronaviruses that infect humans, such as HCoV-OC43, function as the receptor binding domains (RBDs) [33,34]. Recent work showed that the NTD of SARS-CoV-2 may bind cellular receptors other than ACE2 and mediate the entry of the virus into cells that do not express ACE2 [35]. Furthermore, the cell-surface protein neuropilin-1 has been reported to facilitate SARS-CoV-2 cell entry, especially into brain cells [36,37,38], although the mechanism is not fully understood.

The spike proteins of SARS-CoV and SARS-CoV-2 are highly homogenous. They share about 76% sequence identity and have similar domain organizations and similar mushroom-like pre-fusion structures. Superimposition of the two spike protein structures showed a root mean square deviation of 3.8 Å over 959 aligned Ca atom pairs [19]. The SARS-CoV-2 spike protein structure does have differences from the structure of the SARS-CoV spike. An insertion at the S1/S2 protease cleavage site of SARS-CoV-2 S results in an “RRAR” sequence that is recognized by the furin enzyme. Inhibitors of furin significantly reduce SARS-CoV-2 replication in cells, experimentally verifying the importance of the furin cleavage site in the SARS-CoV-2 S protein [39,40].

Structural studies of the soluble SARS-CoV and stabilized SARS-CoV-2 spike protein ectodomains revealed heterogenous conformations, especially among the conformations of CTD1s, which are the RBDs in the S proteins of both viruses. In the S trimers of SARS-CoV and SARS-CoV-2, the RBDs can be in either an “up” or a “down” conformation [19,25,41,42] (Figure 1b). Between one-third and one-half of the spikes have one “up” and two “down” RBDs, whereas the rest of the spikes have all the RBDs in the “down” conformation. Consistent with the studies on the soluble ectodomains, recent cryo-electron tomography (cryoET) studies of the intact SARS-CoV-2 also revealed S trimers with at least one RBD in the “up” conformation, although most had all RBDs in the “down” conformation [43,44]. The “up” angle of the RBD varies in the spikes with the “up” RBD, suggesting that the “up” and “down” conformations might be in dynamic equilibration [42,45]. SARS-CoV-2 spikes with more than one “up” RBD have been observed by cryoET [44] and were also discovered for the Middle East respiratory syndrome coronavirus (MERS-CoV), another coronavirus that causes lethal acute respiratory disease [42]. A recent study of the D614G SARS-CoV-2 mutant S protein showed that the D614G mutation did not alter S protein synthesis, processing, or incorporation into SARS-CoV-2 particles but did result in more than one “up” RBD in trimer spikes. The spikes with more than one “up” RBD may bind more tightly to the host-cell receptor, and this may explain the higher infection rate of this mutant strain [46]. In addition, a functional comparison of the S protein bearing D614 and the S protein bearing G614 with pseudotyped retroviruses revealed that the D614G mutation reduces S1 shedding and enhances the infectivity [47]. Recently, a study on an experimentally engineered D614G SARS-CoV-2 reported similar observations in animal models [48].

**Figure 2:**
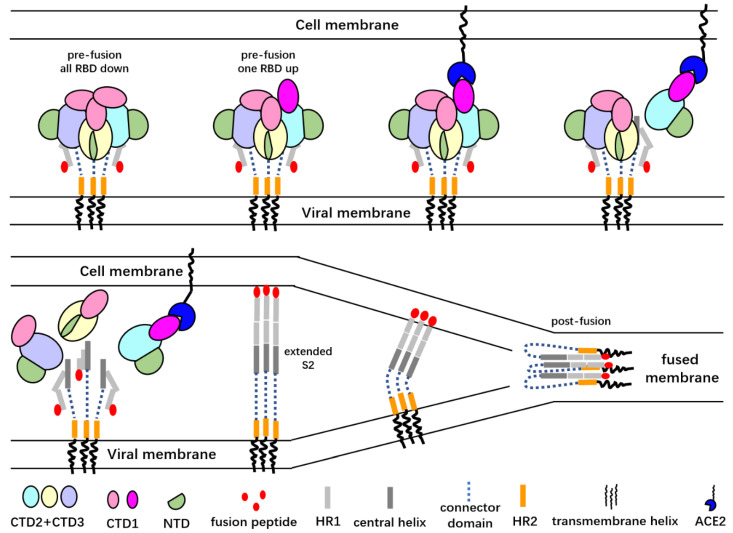
A cartoon representation of the SARS-CoV S glycoprotein-mediated membrane fusion process. Only the “up” RBD is able to bind the angiotensin-converting enzyme 2 (ACE2) receptor, and ACE2 binding promotes the dissociation of the S1 subunits from the trimer spike. Dissociation of the S1 subunits exposes the S2′ cleavage sites and may lead to the extension of S2 subunits and release of the fusion peptide. After the insertion of the fusion peptides into host cell membrane, the α-helical heptad repeat (HR1) regions of the S2 protomers fold back to interact with the HR2 regions to drive the approach of viral and cell membranes. The folded HR1s then interact with HR2s to form a six-helix bundle that brings the viral membrane and host cell membrane close enough to let the membrane fusion happen.

Spikes with more than one “up” RBD are not stable, and the S1 subunits may fall off the spike as has been observed for the spike of MERS-CoV [20,42,45,49,50] (Figure 2). The cryoET studies of the SARS-CoV-2 virion particles showed that a minor portion of the spikes on the viral surface are in the post-fusion state [43,44]. Although the post-fusion state of the spikes may have artificially resulted from sample inactivation and the purification process, this result suggests that the S1 subunits may not be stable and that they are prone to disassociate from the spike under certain conditions. In a recently reported single-particle cryo-electron microscopy structure of the full-length SARS-CoV-2 spike protein with detergent in the buffer, a significant portion of the S trimers were in the post-fusion conformation, and S trimers with one RBD in the “up” state were rare [51]. Most likely the detergent used to stabilize the transmembrane helix of the full-length protein disrupted the hydrophobic interactions between the S1 and S2 subunits and promoted the pre- to post-fusion conformational changes.

Of note, the “up” conformation of CTD1 has been observed so far only in the spikes of viruses that use CTD1 as the receptor binding domain. However, although HCoV-229E uses CTD1 as the receptor binding domain and requires the “up” RBD for binding of its receptor amino-peptidase N (APN), no “up” conformation has been observed for any of the three RBDs [52]. This suggests that the “down” to “up” conformational changes of the RBDs are triggered to occur prior to the binding of the receptor for certain S proteins such as that of HCoV-229E.

## 3. Low-Affinity Binding to Heparin Sulfate Proteoglycans

Before viruses interact with their specific receptors at sites of infection, they attach to the host cell surface and usually “stop” there. Many viral particles are captured by the glycocalyx on the outer surface of the host cell, where the viruses accumulate. Heparan sulfate, which is rich in negative charges, is such a glycosaminoglycan. The binding of heparin to the spike glycoprotein monomer and trimer of SARS-CoV-2 has been detected by surface plasmon resonance [53]. Consistent with this finding, heparin inhibits SARS-CoV-2 infection in a cell culture model [54]. Sequence analysis identified the NTD as the potential glycosaminoglycan binding domain in SARS-CoV-2 S protein [54]. The NTDs of S proteins of other coronavirus bind to different types of polysaccharides or specific polysaccharide receptors [33,55]. The NTD of SARS-CoV-2 also binds lipid molecules, although whether this capacity is relevant to human infection is not known [56]. Binding of the spike to polysaccharides could be important for virus entry even though these molecules are not the specific major receptor, as indicated by a recent study showing that an antibody targeting the NTD of the SARS-CoV-2 S protein neutralizes virus infection [57]. Heparin sulfate proteoglycans are found in many types of human tissues and cells, but SARS coronavirus infection acts in a highly selective manner, which is due to the requirements for specific receptors.

## 4. High-Affinity Binding to the ACE2 Receptor

ACE2 is a type I transmembrane protein consisting of an N-terminal extracellular peptidase domain (PD), a C-terminal collectrin-like domain, a single transmembrane helix, and a short intracellular segment. This protein is expressed on cells of the lung, heart, kidneys, and intestine [58], with high levels of expression in airway epithelial and type I and II alveolar epithelial cells [59]. Functionally, ACE2 negatively regulates the renin–angiotensin system and is involved in amino acid transmembrane transport and uptake [60].

The RBDs of the SARS-CoV and SARS-CoV-2 S proteins are responsible for the interaction with ACE2. The PD of ACE2 interacts with the SARS-CoV-2 RBD mainly through polar residues with nanomolar-level binding affinity [19,41]. It appears that the SARS-CoV-2 S ectodomain has a higher affinity for ACE2 than does the same domain of SARS-CoV, and this may be one reason for the higher infection efficiency of the SARS-CoV-2 [19,41]. Structural studies of both the SARS-CoV and SARS-CoV-2 S protein showed that only the “up” RBD is able to bind ACE2, whereas “down” RBDs are in a receptor-binding-inactive conformation [45]. Binding of ACE2 to one SARS-CoV RBD opens up the RBD (the ACE2-bound RBD has an “up” angle of 51–111 degree compared to 52–70 degrees for the uncomplexed RBD) and induces the release of the S1 subunits from the spikes and the pre- to post-fusion conformation transition of the spike [25]. A two-RBD “up” state and a three-RBD “up” state were observed after ACE2 binding to the S of SARS-CoV-2 [61]. These states are not stable and are prone to switch to the post-fusion state. Serological studies on convalescent patients have found that multiple neutralizing antibodies that target the RBD can compete with ACE2 for interaction with the S protein [62,63,64]. The epitopes of most of these neutralizing antibodies overlap with the ACE2 binding area that is completely exposed only in the “up” RBD. Thus, the “up” RBD conformation of the S trimer may also function in evasion of immune surveillance [65]. Given the sparse decoration of spikes and the small number of “up” RBDs on the virus surface, the titer of RBD-specific neutralizing antibodies after natural infections could be too low to provide protection from a second infection.

It was proposed that the RBD could be used as a vaccine. For SARS-CoV-2, several studies have proven that immunization with the RBD protein subunit or with mRNAs encoding the RBD stimulate a robust immune response and production of a high titer of neutralizing antibodies [66,67,68,69,70]. One group has reported that the proportion of S trimers with 2 or 3 “up” RBDs can be increased through modifying the stabilized pre-fusion S trimer [71]. It will be interesting to see whether this modified S trimer can induce a more potent protective immune response.

Although there are high-resolution structures that provide some details of the interaction between the RBD and either the PD domain or the full-length ACE2 protein [18,72,73], there are still some open and formidable questions regarding receptor binding. ACE2 may exist as a homodimer in tissues where it is co-expressed with the B^0^AT1, an amino acid transporter [72,74]. It was proposed that the ACE2 dimer can accommodate two S protein trimers, although this has not yet been experimentally confirmed. CD26, the molecule that functions as the receptor for MERS-CoV, is a homodimer [75,76,77]. In vitro binding of the ectodomain dimer of CD26 to the spike of MERS-CoV causes aggregation of the spikes and similar complexation may happen for SARS-CoV or SARS-CoV-2 upon binding to the dimer form of ACE2.

## 5. Protease-Mediated Priming of the Spike Protein

The S proteins of SARS-CoV and SARS-CoV-2 are fully or partially proteolytically processed during biogenesis and thus present a ready-to-go S1 fragment on the top of the spike glycoprotein complex on the virus surface. Compared to the SARS-CoV S protein, the S protein of SARS-CoV-2 has an insertion of a furin-like cleavage site at the S1/S2 boundary, which suggests that the SARS-CoV-2 S on the virus surface may have a higher proteolytic processing rate than S from SARS-CoV [78,79]. The S2′ cleavage sites are highly conserved in these two viruses and are completely buried in the pre-fusion state of the S protein [25,42]. Release of the S1 subunits from the S may free the N-terminal fragment of the S2 subunit and expose the S2′ cleavage site [25]. A loss-of-function mutation in the S2′ site prevents the pre- to post-fusion conformation changes of the S protein [25]. These data indicate that cleavage of the S2′ site must occur at an intermediate state after the release of S1 subunits. The endosomal cysteine proteases cathepsin B and L (CatB/L) and the cell-surface protein transmembrane protease serine 2 (TMPRSS2) are involved in proteolytic processing of the S proteins of SARS-CoV and SARS-CoV-2. Complete inhibition of virus infection is observed when target cells are treated with a combination of CatB/L- and TMPRSS2-specific inhibitors [78]. The CatB/L proteases are believed to have optimal activities under acidic pH. TMPRSS2 is located on the cell surface, and clinically approved inhibitors targeting this protease are potential drug candidates for treatment of SARS and COVID-19 [78]. TMPRSS2 is reported to be associated with ACE2 in some cell types. The structure of the complex of these two proteins with and without spike protein will be informative. Details regarding the structures that facilitate the proteolysis of S protein by CatB/L and TMPRSS2 are currently unknown and will also inform drug development. Due to the distinct subcellular locations and environmental pHs of these proteolysis events, the mechanisms of cleavage by CatB/L and TMPRSS2 might be different.

Enveloped viruses enter host cells by two main routes: by direct fusion with the plasma membrane or after receptor-mediated endocytosis with the endosome membrane in a low-pH environment [80]. SARS-CoV and SARS-CoV-2 utilize both routes. The endocytosis route may not be the major one for SARS-CoV entry since the prevention of pH change in endosomes does not considerably reduce infection [81,82]. In addition, unlike other type I fusion proteins such as the HA protein of influenzas virus, low-pH treatment does not induce visible conformational changes in the SARS-CoV spike [25]. Proteases in the extracellular milieu, such as trypsin, thermolysin, and furin, may enhance the preprocessing of the S protein and reduce the dependency of intracellular proteases [83]. Similarly, furin has been shown to cleave at the SARS-CoV-2 spike at the S1/S2 site [65,84]. These might all be hints of the adaptive evolution of coronaviruses.

## 6. Conformational Changes of the Spike Proteins during Virus Entry

Similar to other type I fusion proteins, the pre-fusion state of SARS-CoV spike protein is metastable and transformation into its membrane fusogenic structure occurs readily [20,31]. Structural studies of the SARS-CoV S glycoprotein ectodomain and its complex with ACE2 in several in vitro settings, which attempt to recapitulate the speculated key events during natural infection, found that neither trypsin cleavage nor acidic pH treatment trigger fusogenic conformational changes of the SARS-CoV S glycoprotein. In contrast, the binding of ACE2 to the trypsin- and acidic pH-treated S ectodomain does promote the release of the S1–ACE2 complex and paves the way for the pre- to post-fusion conformational change as indicated by the formation of rosette-like structures of the clustered SARS-CoV S2 trimers [25]. These studies suggest that the disassociation of one S1–ACE2 from the S trimer could induce further disassociation of the other two S1 subunits from the spike trimer. Interestingly, the binding of S230, a potent neutralizing and RBD-targeting monoclonal antibody, in combination with trypsin cleavage of S glycoprotein, triggers fusogenic rearrangements, indicating that antibodies that bind the RBD can mimic receptor binding [50]. Similar observations have also been reported for MERS-CoV [85] and for human immunodeficiency virus [86,87]. These studies suggest that the release of the S1 by receptor or antibody binding is a precondition for coronavirus fusion. Similar observations were also reported for SARS-CoV-2 [61]. Spikes in a post-fusion-like state were observed on the surface of SARS-CoV-2 with electron microscopy [43,44]. It was also reported that a subset of proteins in a post-fusion-like structure are present during the purification of full-length SARS-CoV-2 S glycoprotein [51]. It is still elusive whether the SARS-CoV-2 S protein more readily undergoes transformation into the post-fusion state than S proteins of SARS-CoV or MERS-CoV.

Following priming, conformational change in the S2 subunits, which are buried under the S1 subunits, expose the fusion peptide to the target membrane [88,89,90]. Several intermediate states may exist during this conformational change (Figure 2). The nine-helix core of the S2 stem, like those of other type I fusion viral proteins, may undergo conformational changes to form an intermediate extended stalk after receptor binding (Figure 2). It is still not clear whether the release of the S1 subunits is required prior to the formation of the extended stalk. Nevertheless, even for viruses that do not use CTD1 as the receptor binding domain, the “down” to “up” conformational changes of the CTD1s must be triggered to allocate space for the formation of the extended stalk [25]. The signal for triggering the “down” to “up” transformation in the S trimer has not been determined. The exposure of the fusion peptide requires cleavage at the S2′ site. The cleavage may occur either before or after the formation of the extended stalk. Cleavage prior to the extension of the central helices requires extracellular proteases. The advantage of cleavage that occurs after the extension of the central helices is that the cleavage sites are located at the distal end of the stalk, close to the cell-surface-anchored proteases [25].

Following the cleavage and exposure of the fusion peptides, the fusion peptides are inserted into the host cell membrane. In order to drive the fusion of virus and host cell membranes, the α-helical heptad repeat 1 (HR1) region of the S2 protomer folds back to interact with the HR2 region [91]. The folded HR1s then interact with the HR2s to form a six-helix bundle to bring the viral membrane and host cell membrane close enough together to allow membrane fusion to happen [90] (Figure 2). Observations of the post-fusion-like spikes on the viral surface and in detergent-solubilized full-length spikes suggest that the insertion of the fusion peptide into the host cell membrane might be a rate-limiting step in the entry procedure [43,51]. The post-fusion-like spike may be formed by fold back of the extended S2 without successful insertion into host cell membrane. All these steps, from receptor recognition and binding to protease-mediated priming to S2 conformational changes and finally to membrane fusion, are precisely regulated.

The viral membrane fusion process may be one of the most sophisticated phenomena in nature, involving complex and rapid conformational changes of the fusion protein and the lipid bilayers that are hard to examine. Most of our hypotheses about the process are based on the comparison of the pre-and post-fusion structures. However, due to the difficulties in preparing homogenous samples suitable for structure determination, most of the intermediate states have not been observed. More studies are needed to confirm the intermediate states, especially of native virus, cell surfaces, or endosomes. An accurate understanding of the structural basis of SARS-CoV and SARS-CoV-2 membrane fusion will be invaluable in drug discovery and vaccine development.

## 7. Inhibition of Entry as a SARS and COVID-19 Treatment

Benefiting from our current understanding of SARS coronavirus entry, vaccines, antibodies, and small-molecule drugs are being developed for clinical use. Every step during virus entry into the cell and of virus replication and packaging could, in principle, be the target of inhibitors. For example, as mentioned above, heparin inhibits the attachment of coronavirus onto the surface of cells. Recently, an engineered, soluble, and catalytically active ACE2 with higher binding affinity to SARS-CoV-2 S protein than the natural receptor has been produced and tested as a decoy receptor to limit virus infection [92]. A number of neutralizing antibodies that target the RBD or other regions of the spike have been discovered that interfere with receptor binding to the S protein; some are currently being tested in clinical trials. Chloroquine and hydroxychloroquine, which change the intracellular pH have inhibitory activity against SARS-CoV-2 infection in certain cell types [93]. A clinically proven TMPRSS2 inhibitor has been reported to be highly efficient in the inhibition of SARS-CoV-2 infection of lung cells [78]. Combined inhibitors specific for furin and TMPRSS2 have shown promising results in the inhibition of virus propagation in respiratory tissue [39]. Peptides based on the HR2 sequence have been designed that interfere with membrane fusion [94]. Upon the release of the viral genome into the cytoplasm, inhibitors targeting virus-encoded enzymes, such as the papain-like protease, 3-chymotrypsin-like protease, helicase, RNA-dependent RNA polymerase, and primase, have the potential to inhibit virus replication [95]. Inhibitors targeting the early steps of virus infection could be used as preventive measures. These types of drugs as well as vaccines are urgently needed to stop the global COVID-19 pandemic. Further mechanistic studies that reveal molecular details of the entry of SARS coronavirus into host cells will guide the development of additional antiviral therapies.
